# Cold Plasma-Activated Water in Food Processing: Process Engineering, Mechanistic Insights, and Sustainability Perspectives

**DOI:** 10.3390/foods15213831

**Published:** 2026-10-26

**Authors:** Mustapha Abdullateef, Mustapha Muhammad Nasiru, Cunshan Zhou, Chunyang Li, Ghulam Mustafa, Evans Frimpong Boateng, Xiaojie Yu

**Affiliations:** 1 School of Food and Biological Engineering, Jiangsu University, Zhenjiang 212013, China; 2 Department of Food Science and Technology, Bayero University, Kano 700006, Nigeria; 3 College of Food Science and Engineering, Bohai University, Jinzhou 121013, China; 4 Institute of Agro-Products Processing, Jiangsu Academy of Agricultural Sciences, Nanjing 210014, China; 5 State Key Laboratory for Quality and Safety of Agro-Products, Institute of Agro-Product Safety and Nutrition, Zhejiang Academy of Agricultural Sciences, Hangzhou 310021, China; 6 Department of Food Science and Technology, School of Agricultural and Technology, University of Energy and Natural Resources, Sunyani P.O. Box 214, Bono, Ghana

**Keywords:** cold plasma activated water, non-thermal food processing, reactive oxygen and nitrogen species, food safety, process optimization, sustainability

## Abstract

Growing demand for safe, sustainable, and minimally processed foods has increased interest in emerging non-thermal preservation technologies capable of reducing environmental impact while maintaining nutritional and sensory quality. Among these technologies, cold plasma-activated water (CPAW) has gained considerable attention as an eco-efficient and versatile processing medium for food applications. CPAW is generated through the dissolution of plasma-induced reactive oxygen and nitrogen species (RONS) in water, producing a chemically active solution with antimicrobial, oxidative, and enzyme-modulating properties. Depending on its reactive-species composition and storage conditions, CPAW may retain activity for delayed application, offering greater operational flexibility than direct plasma treatment. Building on established plasma–liquid applications in water purification, this review critically examines current knowledge on the generation of CPAW, its physicochemical characteristics, and mechanisms of action, with emphasis on its interactions with microorganisms, food macromolecules, and enzymes. It also covers recent advances in analytical characterization, application strategies, safety assessment, and sustainability considerations, discussing how plasma operating conditions, gas composition, and storage parameters shape CPAW stability and performance. The review highlights the limitations of treatment-time-based reporting and the need for standardized physicochemical dose descriptors to improve reproducibility and cross-study comparison. Although CPAW offers potential as a low-residue technology with promising sustainability advantages, challenges related to mechanistic understanding, scalability, process standardization, regulatory acceptance, and consumer acceptance remain significant barriers to industrial adoption. Addressing these limitations through interdisciplinary research and technological optimization will be necessary for the successful integration of CPAW into next-generation sustainable food-processing systems.

## 1. Introduction

The food-processing industry is currently undergoing a significant shift toward eco-friendly, sustainable, and non-thermal processing technologies. The increasing consumer awareness about nutrition, health, and environmental sustainability has translated into a high market demand for minimally processed foods with improved safety and quality. Traditional preservation methods, such as chemical preservatives, thermal sterilization, and synthetic additives, although effective, have been increasingly criticized due to their adverse effects on quality, nutrient and environmental sustainability [1,2].

The widely used chemical preservatives (sodium hypochlorite, chlorine dioxide, quaternary ammonium compounds, etc.) are of serious concern. These chemicals generate toxic chemical residue, thereby leading to chemical pollution of aquatic systems, and posing challenges related to occupational safety [3]. More critically, repeated or sublethal exposure to certain disinfectants has raised concerns regarding possible selection or co-selection of tolerant microbial populations and potential links with antimicrobial resistance. There is therefore a need to develop residue-minimizing, effective, and environmentally sustainable alternatives.

Among the novel emerging techniques, cold plasma (CP) has gained attention due to its high antimicrobial efficacy and ability to modify food functionality without significant heat input [4,5]. CP technology employs a non-thermal electrical discharge to generate a partially ionized gas containing energetic electrons, ions, neutral species and reactive oxygen and nitrogen species (RONS), generally while maintaining a relatively low bulk gas temperature [5,6]. The reactive species (hydrogen peroxide, ozone, hydroxyl radicals, and nitric oxides) interact with the microbial cell membranes, enzymes, and biomolecules, thereby leading to microbial inactivation and structural modifications within food matrices [6,7]. The flexibility of CP and its ability to be used at low temperatures make it a suitable option for improving food safety and extending shelf life while preserving nutritional quality.

A notable indirect application of CP is the generation of plasma-activated water. Beyond food processing, plasma–liquid technologies have been extensively investigated for the purification of water and wastewater treatment, particularly for microbial inactivation and the degradation of organic contaminants [8,9]. In food processing, the terms plasma-activated water (PAW) and cold plasma-activated water (CPAW) are often used interchangeably. In this review, CPAW is used specifically to refer to water activated through non-thermal plasma systems under controlled operational conditions. Also, since much of the published literature uses the broader term PAW, studies described as PAW were interpreted within the CPAW framework only when non-thermal operation could be established from the reported plasma source, discharge configuration, operating conditions, temperature information, or the authors’ explicit description of the system as cold or non-thermal plasma. Where the information reported was insufficient to establish non-thermal operation, the study was not used as CPAW-specific quantitative evidence and was considered only within the broader plasma–liquid context where relevant. CPAW is characterized by the presence of RONS and also by its improved control over reactive species composition, physicochemical stability, concentration, and flexibility [7,10]. 

CPAW is produced when water is exposed to cold plasma, leading to the dissolution of long- and short-lived reactive species such as hydrogen peroxide, nitrate, nitrite, ozone, hydroxyl radicals, and peroxynitrite [7,10]. The species generated in CPAW provide antimicrobial activity and regulate enzyme function through controlled oxidation. These effects enable the CPAW to reduce microbial contamination and quality deterioration while remaining easy to apply as a liquid, leaving little or no harmful residue after treatment. Unlike direct cold plasma treatment, CPAW separates plasma generation from its application, enabling the activated water to be generated in advance, transported, and applied when needed. This separation may facilitate integration into existing processing operations, although reliable industrial scale-up remains challenging because of RONS stability, treatment uniformity, reactor design, and process-control requirements. 

Mechanistically, CPAW exerts its preservative effects through the RONS generated during plasma activation. These species inactivate microorganisms and induce controlled oxidative modifications of food components, including proteins, starches, and lipids. Consequently, CPAW enhances microbial safety, extends shelf life and also influences the nutritional, structural, and functional properties of foods [10,11]. CPAW accomplishes these effects and also minimizes nutrient degradation as commonly observed during thermal processing, aligning well with contemporary green processing principles.

Despite these advantages, significant knowledge gaps remain regarding the mechanisms, physicochemical characterization, and standardization of CPAW. The stability and reactivity of CPAW are highly influenced by operating parameters of the plasma (e.g., power input, gas composition, discharge configuration), treatment duration, and storage conditions [7,10,12]. As a result, reproducibility across studies remains limited, and the long-term safety of CPAW-treated foods requires further evaluation. Moreover, the relationship between CPAW and complex food matrices, particularly those rich in lipids, proteins, and phenolic compounds, presents additional challenges that warrant systematic investigation.

Recent studies in CPAW have shown its potential as a versatile non-thermal technology for food processing and preservation. Although numerous studies have investigated its antimicrobial efficacy, knowledge of its physicochemical properties, mechanisms of action, interactions with food components, and practical applications remains fragmented. Bringing these studies together is essential for adequately understanding the current state of the technology and identifying the areas that need further research. This review, therefore, critically examines the generation of CPAW, the factors influencing its physicochemical characteristics, and the mechanisms through which it interacts with microorganisms, enzymes, and major food macromolecules. It also discusses current and emerging applications in food processing, analytical approaches used to characterize CPAW, and the challenges associated with process optimization, industrial implementation, and regulatory acceptance. This review adopts an integrated engineering perspective by relating plasma generation conditions to CPAW properties, molecular interactions, food matrix responses, process scalability, sustainability, and regulatory considerations. By highlighting both recent advances and existing knowledge gaps, the review provides a framework for future research and supports the development of CPAW as a practical technology for sustainable food processing.

## 2. Cold Plasma and CPAW

### 2.1. Fundamentals

Cold plasma (CP) has been one of the viable alternatives to conventional chemical-intensive processing, largely because it generates an array of reactive species at or near room temperature, potentially mitigating the need for conventional chemical and high-temperature treatments. Also referred to as non-thermal plasma, it consists of a partially ionized gas containing electrons, ions, ultraviolet photons, and RONS, which can drive chemical transformations while maintaining the processed material at a relatively low temperature [10,12].

Unlike thermal plasma, cold plasma systems can operate at atmospheric, near-atmospheric or low pressure depending on the discharge configuration, thereby generating high-energy electrons with a low bulk gas temperature, making it well-suited for treating temperature-sensitive food materials [5,13]. The discharge configuration, including dielectric barrier discharge (DBD), corona discharge, cold plasma torches or jets, and gliding arc discharge, together with the working gas (e.g., air, oxygen, nitrogen or argon), influences plasma chemistry and the reactive species transferred to water [7,10]. These parameters can be adjusted according to the intended food application.

**Figure 1:**
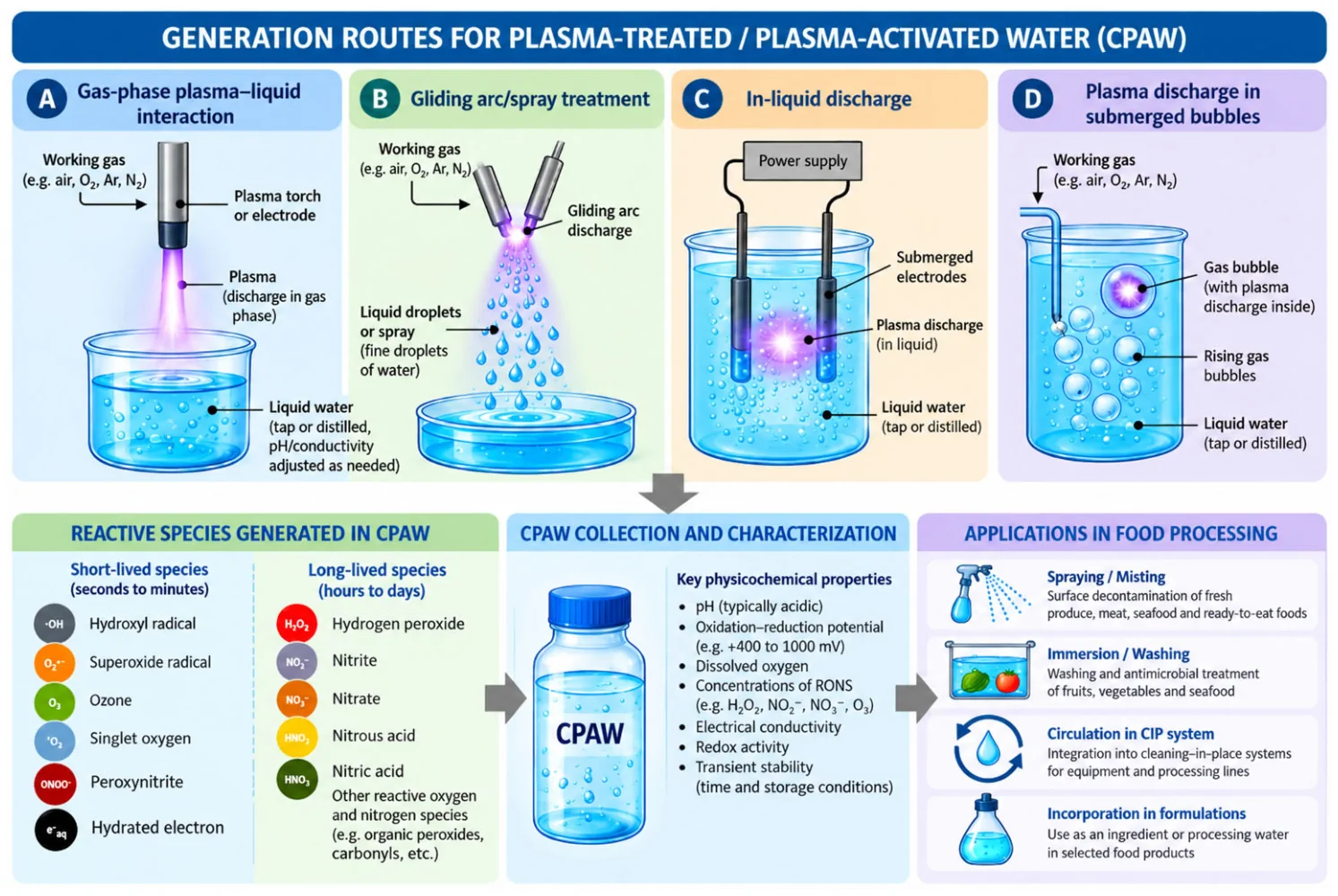
Representative plasma–liquid configurations for the generation of plasma-treated and plasma-activated water: (**A**) gas-phase discharge interacting with a liquid surface; (**B**) gliding arc or related discharge interacting with liquid droplets or sprays; (**C**) discharge initiated within liquid using submerged electrodes; and (**D**) plasma generated within gas bubbles dispersed in water.

When cold plasma interacts with water, it gives rise to CPAW, an indirect application of cold plasma technology. Production of CPAW occurs through treatment of food-grade water, deionized water, or even pure water with cold plasma, leading to the formation of reactive species such as hydrogen peroxide (H_2_O_2_), ozone (O_3_), hydroxyl radicals (•OH), nitric oxide (NO), nitrite (NO_2_^−^), and nitrate (NO_3_^−^) [12,14]. This aqueous medium acts as a carrier of plasma-generated reactive species, enabling indirect but effective application in food processing. Figure 1 presents a pictorial summary of CPAW generation, reactive species formation, and food-processing applications.

One of the main advantages of CPAW compared to direct plasma treatment is its compatibility with standard food handling procedures, such as dipping, spraying, and washing. This feature enables the integration of CPAW into existing sanitation lines without repositioning high-voltage plasma reactors near the food itself [10]. Furthermore, CPAW provides more uniform exposure and reduces geometric shadowing effects inherent in direct plasma applications, and ensures thorough surface exposure in complex food matrices [10,12].

The microbial and enzyme inactivation efficacy of CPAW is mainly attributed to the oxidative activity of its reactive species [7,15]. In addition, the acidity of CPAW, resulting from the formation of nitric and nitrous acids, contributes to its antimicrobial activity [2,15]. However, optimization of the treatment conditions is greatly needed to minimize undesirable effects on the quality of food [2].

Recent studies have shown that CPAW can alter both the biochemical and structural characteristics of food constituents. It has been reported that CPAW treatment can induce protein conformational changes, influence structural strength in starch granules, and enhance bioactive compound extraction through cell disruption [10,16]. These findings suggest the potential applications of CPAW in value-added processing besides the preservation of foods.

Given the growing importance of CPAW in food applications, understanding its physicochemical properties is essential for evaluating its performance. The following section examines the principal physicochemical characteristics of CPAW and the analytical approaches used for its characterization, which are fundamental to understanding its antimicrobial performance and interactions with food systems.

#### Plasma-Based Water Treatment and Its Relationship with CPAW Generation

Plasma-based water treatment represents an important application of plasma–liquid interactions and provides a broader technological context for the development of CPAW. Electrical discharges are employed during water purification and wastewater treatment to generate reactive species that contribute to the inactivation of microorganisms and the degradation or transformation of chemical contaminants [8,9]. Depending on reactor design and operating conditions, these species may be generated in the gas phase and subsequently transferred into water, formed at the plasma–liquid interface, or produced through electrical discharges initiated within the liquid [9]. Plasma-based systems have been investigated for the treatment of water containing microorganisms, pesticides, pharmaceutical residues, and other organic pollutants [8].

Plasma–liquid systems are classified by where the discharge is generated relative to the liquid: above the surface, at the gas–liquid interface, directly submerged, within bubbles, or in contact with dispersed droplets and sprays [9,17]. In surface-treatment systems, plasma-generated species interact with the liquid through the gas–liquid interface. In submerged systems, electrical discharges may develop in vapor or gas cavities formed around immersed electrodes, while externally supplied gas bubbles can provide an additional discharge medium [9]. Bubble-assisted configurations increase the plasma–liquid interfacial contact area and can facilitate the transfer of reactive species into the surrounding liquid. Gliding arc systems have also been investigated for liquid treatment, including arrangements in which water droplets or sprays interact with the discharge [9,18,19].

Although plasma-based water purification and CPAW generation share fundamental plasma–liquid chemistry, their treatment objectives are different. Water purification primarily aims to remove or transform undesirable contaminants and reduce microbial contamination [17]. By contrast, CPAW generation seeks to produce water with a controlled reactive-species composition for subsequent application to foods, food-contact surfaces, or processing operations [7,10,20]. Therefore, effective contaminant removal does not automatically establish that the resulting water is suitable for food processing. Its microbiological and chemical quality, residual reactive species, possible transformation products, and compatibility with the intended food application must be assessed.

This distinction is particularly relevant to process engineering. Reactor configurations developed for water treatment provide useful knowledge concerning discharge formation, reactive-species generation, gas–liquid mass transfer, energy efficiency, and scale-up. Nevertheless, the optimal operating conditions for contaminant degradation may differ from those required to generate reproducible CPAW while maintaining food safety and quality. Accordingly, the selection of a plasma reactor should consider both the intended treatment objective and the physicochemical characteristics required of the treated water.

### 2.2. Physicochemical Properties and Characterization of CPAW

The performance of CPAW is largely determined by its key physicochemical properties, such as pH, oxidation–reduction potential (ORP), electrical conductivity (EC), and reactive species composition, all of which influence its antimicrobial efficacy and biochemical interactions. The initial quality of the water used for plasma activation is another important determinant of CPAW composition. Unlike experiments conducted with deionized or distilled water, industrial food-processing water may contain dissolved minerals, organic matter, and other constituents that influence plasma-induced reactions and reactive-species persistence [21]. Therefore, the water-treatment objective and the water-activation objective should not be conflated. Where plasma is used to purify contaminated water, the treated water must be evaluated against the relevant requirements for its intended use. Where the objective is CPAW generation, the initial water quality and resulting reactive-species composition should both be reported to support reproducibility and food-safety assessment.

As illustrated in Table 1, CPAW has been reported to exhibit acidic pH values due to the formation of nitric and nitrous acids; however, the magnitude of acidification depends strongly on feed gas, discharge configuration, activation time, and initial water chemistry. This acidic environment enhances antibacterial activity and contributes to enzyme suppression; generally, lower pH values are attributed to stronger antimicrobial effects [20,22]. The ORP under a CPAW treatment has been reported to increase during plasma activation, with values varying according to treatment [20,23]. However, direct comparisons among studies require caution because plasma conditions, water composition, and measurement protocols differ. This high ORP is mainly associated with relatively stable reactive species such as hydrogen peroxide, nitrate, and nitrite, which significantly contribute to the antimicrobial activity of CPAW [6,22,23]. An increase in EC reflects the accumulation of plasma-generated ionic species in CPAW. Consequently, EC provides an indirect indication of changes in dissolved ionic species but should be interpreted alongside direct RONS measurements when assessing CPAW composition and storage stability [20,21,23]. The concentration and availability of key reactive species, including H_2_O_2_, NO_2_^−^, O_3_, and •OH, strongly influence antimicrobial activity, enzyme inactivation, and biochemical modifications in food systems [20,22,23,24].

The storage stability of CPAW remains an important limitation because short-lived species such as ozone and hydroxyl radicals decay rapidly, while longer-lived species such as nitrate and nitrite persist for extended periods [7,20,25]. However, their long-term stability and regulatory safety still require further evaluation [10]. Additionally, a slight reduction in surface tension improves wetting efficiency, which can enhance surface contact and improve decontamination performance [26]. Overall, these physicochemical properties strongly influence the functionality of CPAW and highlight the importance of detailed characterization for achieving stable and efficient food-processing applications.

The breadth of the reported physicochemical ranges under different CPAW treatments highlights a fundamental reproducibility problem. Solutions described using the same term, CPAW, may differ in pH, ORP, conductivity, and RONS concentrations. Consequently, CPAW used in one study may not be chemically equivalent to CPAW used in another, complicating the interpretation of differences in antimicrobial and quality outcomes. These variations highlight the significance of standardized reporting, as discussed in the following subsection.

**Table 1:** Key physicochemical properties of CPAW and their relevance to food processing.

Property	Reported Values/Ranges Under Cited Experimental Conditions	Influencing Plasma Parameters	Functional Role in Food Processing	Relevant Applications	References
pH	2.0–3.5 (acidic)	Feed gas type & rate, input power, treatment time	Acidification enhances microbial inhibition and enzyme inactivation	Surface sterilization, fruit/vegetable wash	[20,22,24]
Oxidation-Reduction Potential (ORP)	420 to 580 mV	Discharge power, exposure time, gas composition	Drives redox reactions for pathogen inactivation	Inactivating bacterial biofilms, fungi, and spores	[20,22,23,27]
Electrical Conductivity	0.5–3.0 mS/cm	Treatment time, volume, discharge voltage and power	Indicates ion content (NO_3_^−^, NO_2_^−^), linked to antimicrobial potential	Dairy cleaning, sanitation of equipment	[20,23,28]
Reactive Species (e.g., H_2_O_2_, NO_x_, OH•, O_3_)	H_2_O_2_: Up to 16–218 mg/L; NO_2_^−^: Up to 80–858 mg/L; NO_3_^−^: 650 mg/L; O_3_, OH• present	Plasma type, power, time, gas, storage	Involved in microbial inactivation, biochemical modifications, oxidizes lipid membranes and degrades surface agrochemicals	Juice preservation, enzyme deactivation	[22,23,24,29,30]
Shelf Stability	1–14 days (depends on storage)	Storage temp, light exposure	Limits off-site or delayed applications,	On-site treatment systems	[10,31]
Surface Tension	Decreased slightly	Gas species, plasma configuration	enhances wetting and surface coverage on foods	decontamination, emulsions, agriculture	[5,10,12,26]

Note: Reported values should not be interpreted as universal ranges for CPAW. Physicochemical properties vary substantially with plasma source and configuration, feed gas, discharge power, activation time, plasma–liquid geometry, initial water composition, water volume, temperature, analytical method, and storage conditions. Values shown summarize observations from the cited studies and are intended to illustrate the breadth of reported CPAW characteristics rather than define standardized specifications.

#### Minimum Reporting Requirements for CPAW Studies

The variability in CPAW generation conditions and physicochemical characteristics highlights the need for standardized reporting to improve reproducibility and facilitate meaningful comparisons across studies. Treatment duration alone is insufficient to describe CPAW dose, as similar exposure times may produce substantially different reactive-species concentrations and biological effects. Therefore, a minimum reporting framework covering plasma generation, CPAW characterization, application conditions, and treatment outcomes is recommended.

For plasma generation, studies should report the plasma source and reactor configuration, applied voltage or discharge power, frequency where applicable, feed gas composition and flow rate, plasma–liquid distance or contact geometry, and activation time. For water and CPAW characterization, essential information should include the initial water source and composition, water volume, temperature, pH, oxidation–reduction potential (ORP), electrical conductivity, and concentrations of major long-lived reactive oxygen and nitrogen species, particularly H_2_O_2_, NO_2_^−^, and NO_3_^−^. The interval between CPAW generation and application, together with storage temperature and light exposure where relevant, should also be documented. For food application, studies should specify the food matrix, sample mass or surface area, CPAW-to-food ratio, treatment method, contact time, and treatment temperature. For microbiological evaluation, the target microorganism and strain, initial inoculum, microbial recovery and enumeration methods, detection limits, and basis for calculating log reductions should be reported. Where enzymatic activity or food quality is evaluated, the corresponding analytical methods and outcome measures should also be described.

These reporting requirements would improve the interpretation of CPAW efficacy, help distinguish differences in treatment intensity from food-matrix effects, and support the development of reproducible, application-specific dose–response relationships. Although a universally validated CPAW dose metric has not yet been established, consistent reporting of these parameters would provide a practical foundation for process optimization, cross-study comparison, and eventual industrial standardization.

### 2.3. Mechanisms of Action of CPAW on Microorganisms and Biomolecules

**Figure 2:**
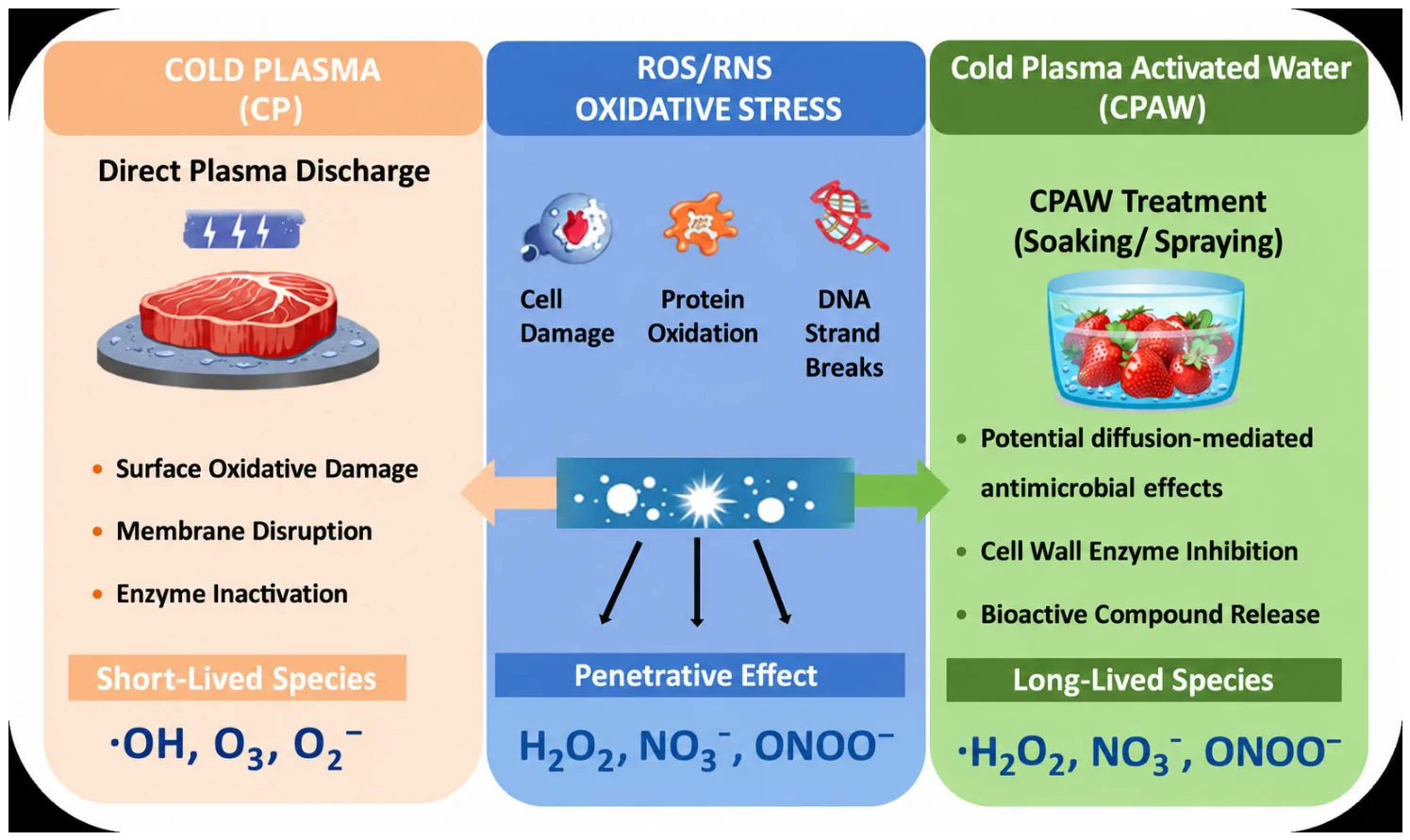
Comparison of action mechanisms between cold plasma (CP) and cold plasma-activated water (CPAW) in food systems. Direct CP primarily induces surface-localized oxidative damage through short-lived reactive species, whereas CPAW enables diffusion-mediated delivery of longer-lived RONS into food matrices, allowing potential diffusion-mediated antimicrobial effects, enzyme inhibition, and internal quality modulation. Both pathways converge via ROS/RNS-mediated oxidative stress mechanisms.

Figure 2 below illustrates the fundamental differences between the microbial inactivation process during direct CP and CPAW treatment, particularly in terms of reactive species lifetime, penetration depth, and mode of action. CP primarily induces surface-localized oxidative damage; CPAW, on the other hand, relies on diffusion-mediated delivery of longer-lived RONS across wetted surfaces and accessible subsurface regions, potentially extending antimicrobial action beyond the immediate treatment interface [7,10,32]. The effectiveness of CPAW is largely associated with the generation of RONS during plasma–water interactions. During the interaction, various short-lived species (•OH, O_2_•^−^, O_3_, ONOO^−^) and longer-lived species (H_2_O_2_, NO_2_^−^, NO_3_^−^) are generated, and these species are attributed to the antimicrobial, biochemical, and biological effects of CPAW [15,32]. 

#### 2.3.1. Microbial Inactivation and Cellular Responses

**Figure 3:**
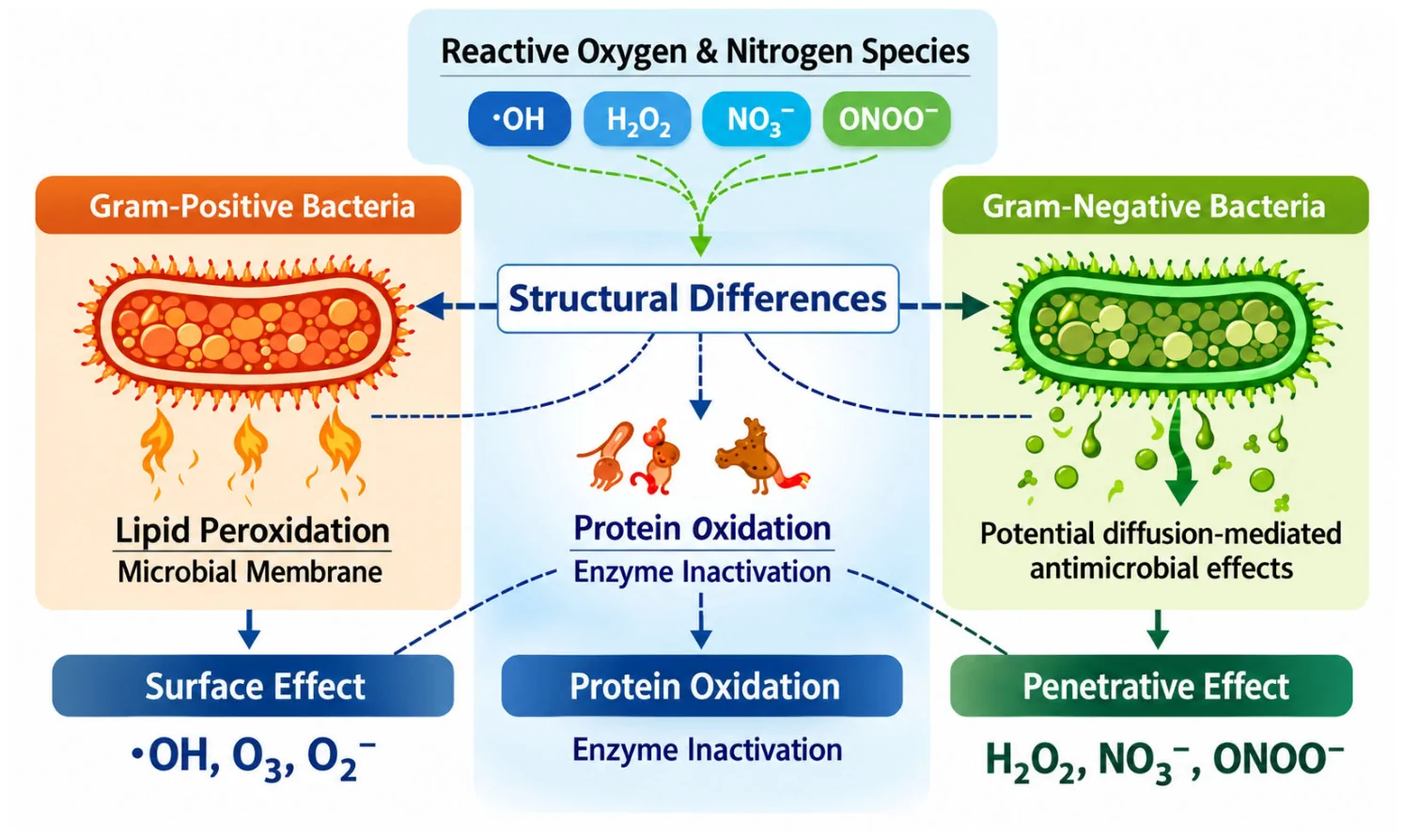
Molecular pathway of microbial inactivation induced by CPAW. Reactive oxygen and nitrogen species initiate lipid peroxidation of microbial membranes, followed by oxidative denaturation of intracellular proteins and enzymes, and ultimately DNA strand breakage. Structural differences between Gram-positive and Gram-negative bacteria influence sensitivity to CPAW treatment.

Oxidative stress is the primary driver of microbial inactivation by CPAW. The generated RONS can interact with microbial cells and cause lipid peroxidation, leading to structural disruption of the cell membrane. This leads to the loss of the structural integrity of the membrane, disruption of transmembrane potential, and increased membrane permeability [6,11,15]. The principal microbial inactivation pathways induced by CPAW are summarized in Figure 3.

Once the structural integrity of the membrane is affected, the intracellular macromolecules (proteins, enzymes, and DNA) become highly susceptible to oxidative attack. This leads to enzyme inactivation, metabolic disruption, and DNA damage [11,13]. The RONS-induced DNA strand breaks, oxidative base damage, and nucleotide modifications can further disrupt cellular replication and DNA repair processes, ultimately leading to irreversible cellular death [7,11,15]. Long-lived reactive species such as H_2_O_2_, nitrite, and nitrate continue to exert antimicrobial effects even after active plasma treatment has ended, thereby extending the antimicrobial action of CPAW.

The antimicrobial action of CPAW should therefore be regarded as a multi-species and condition-dependent process rather than the effect of a single dominant oxidant. Short-lived species such as •OH can initiate rapid, highly localized oxidation during or immediately after plasma–liquid interaction, whereas longer-lived species such as H_2_O_2_, NO_2_^−^ and related nitrogen species can sustain oxidative and nitrosative activity after plasma generation has ceased [7,28,33]. Their effects may also be non-additive: acidification can alter reactive-species speciation and microbial stress tolerance, while interactions among oxygen- and nitrogen-derived species may generate secondary oxidants with different reactivities [11]. However, the relative contribution of each pathway remains difficult to resolve because changing plasma conditions usually alters several RONS simultaneously. Thus, reports of ‘synergistic RONS action’ should be interpreted as evidence of combined chemical effects unless individual species have been experimentally isolated, scavenged, or reconstructed at controlled concentrations.

Microbial susceptibility to CPAW varies depending on cell wall structure. Gram-negative bacteria such as *Escherichia coli* are generally more susceptible because of their thinner peptidoglycan layer compared with Gram-positive bacterial and fungal spores [7,10,15,22]. Across cell types, inactivation broadly follows a sequential inactivation pathway: oxidative stress → membrane damage → intracellular dysfunction → irreversible cell death. Nevertheless, cell-wall classification alone does not fully explain CPAW susceptibility. Differences in strain-specific antioxidant defense, physiological state, initial microbial concentration, biofilm formation, and the surrounding food matrix may alter resistance to oxidative stress. Consequently, apparently inconsistent responses among Gram-positive and Gram-negative organisms across studies should not automatically be attributed to peptidoglycan thickness alone.

At sub-lethal concentrations, however, RONS may function as signaling molecules rather than purely oxidative agents. In such cases, CPAW can trigger Ca^2+^ influx across the cell membrane, activating calcium-dependent signaling pathways involved in antioxidant defense, phenylpropanoid metabolism, and cell wall remodeling [5,7]. These adaptive responses are particularly relevant in plant-based food systems, where controlled oxidative stress may induce beneficial biochemical changes, improve stress tolerance, and enhance certain quality attributes of food materials.

#### 2.3.2. Enzyme Inactivation

Beyond microbial targets, CPAW can effectively modulate food-associated enzymes responsible for quality degradation. Enzymes such as polyphenol oxidase (PPO), peroxidase (POD), and lipoxygenase (LOX) undergo oxidative modification when exposed to RONS. These reactive species interact with sulfhydryl groups and aromatic amino acid residues that are essential for catalytic activity, leading to protein unfolding, loss of active-site integrity, and irreversible enzyme inactivation [10,34,35]. In PPO, residues such as histidine and tyrosine, which are central to catalytic activity, are preferentially attacked by hydroxyl radicals and singlet oxygen, thereby suppressing enzymatic browning without the heat-related damage associated with thermal processing [36]. Experimental studies have reported significant reductions in enzyme activity accompanied by changes in α-helix and β-sheet structures, supporting the hypothesis that enzyme inactivation results primarily from structural disruption rather than pH effects alone [35,36]. Selective enzyme inactivation helps reduce enzymatic browning and oxidative deterioration while minimizing the nutrient losses commonly associated with thermal processing.

#### 2.3.3. Modification of Biopolymers

Food biopolymers such as starches and proteins are also susceptible to structural modification by CPAW-generated reactive species. Chain scission within starch molecules alters gelatinisation and pasting behavior, opening possibilities for developing modified starches suitable for clean-label formulations without recourse to conventional chemical modification [37]. Proteins exhibit different structural responses depending on CPAW treatment intensity. Mild treatments may induce partial unfolding, revealing hydrophobic residues and reactive groups that enhance water solubility, emulsification, or foaming properties [10,37]. More intense exposure may trigger aggregation due to intermolecular cross-linking. These controlled structural modifications may provide functional advantages in developing plant-protein ingredients with improved mouthfeel, digestibility, and processing performance.

### 2.4. Applications of CPAW in Food Processing

The physicochemical properties of CPAW enable its application across a wide range of modern food-processing systems. CPAW is particularly suitable for high-value and heat-sensitive foods because of its non-thermal, low-residue, and energy-efficient mode of action [2,38]. Table 2 summarizes major food categories in which CPAW has been tested, detailing target outcomes, observed effects, and treatment parameters. These reported applications demonstrate the CPAW’s potential for quality preservation, microbial control, and sanitation under the investigated experimental conditions. However, most reported outcomes should be interpreted in relation to their experimental scale, and laboratory efficacy should not be considered equivalent to validated industrial performance. Commercial implementation requires further evaluation of treatment throughput, process consistency, equipment integration, food-quality effects, and compliance with applicable safety requirements.

#### 2.4.1. Microbial Decontamination

Microbial inactivation remains the most extensively studied application of CPAW. CPAW has been reported to achieve > 1 log reduction in fresh produce such as lettuce, strawberries, and blueberries while maintaining desirable sensory attributes [39,40,41]. In meat and poultry systems, >3 log reductions in Salmonella and *E. coli* have been reported with minimal lipid oxidation under optimized conditions [42,43]. Similar benefits have been observed in seafood, where CPAW has been reported to cause reduction in spoilage microorganisms [43,44,45,46]. However, antimicrobial efficacy varies with food-matrix characteristics, treatment conditions, and the target microorganism [26,47]. Rough or porous surfaces of vegetables and fruits may protect microorganisms within crevices and reduce their exposure to RONS, whereas differences in surface composition and organic load can promote rapid scavenging of reactive species [6,47]. These factors should be considered when interpreting microbial reductions reported across different food systems.

#### 2.4.2. Enzyme Inactivation

Beyond microbial control, CPAW effectively modulates enzymatic activities that contribute to browning and degradation. CPAW can inhibit polyphenol oxidase (PPO) and peroxidase (POD) activities by 40–70% in several fruits and vegetables, supporting color retention and quality preservation [5,20,48]. The underlying mechanism involves oxidative modification of thiol groups and alterations in secondary and tertiary enzyme structure, including decreases in α-helix content and increases in β-sheet components [49]. This dual capability (microbial reduction and enzyme control) widens the potential application of CPAW considerably. Nevertheless, the reported range in enzyme inhibition also illustrates substantial inter-study variability. Differences in enzyme source, food matrix, initial enzyme activity, CPAW chemistry, exposure time, and assay methodology can substantially influence the apparent degree of inactivation [5,49]. In addition, a treatment sufficiently intense to suppress PPO or POD may simultaneously oxidize pigments, phenolics, or other quality-related constituents. Therefore, greater enzyme inactivation should not automatically be interpreted as superior process performance. The optimum treatment represents a balance between suppressing undesirable enzymatic activity and minimizing oxidation of desirable food components. Direct comparison among studies remains difficult where CPAW physicochemical properties and residual enzyme activity are reported using different experimental protocols.

#### 2.4.3. Shelf-Life Extension and Quality Retention

Reductions in microbial load and enzymatic degradation translate into improved shelf life and preserved sensory quality. CPAW-treated produce, such as strawberries, bell peppers, and mushrooms, frequently exhibit better firmness, color stability, and lower lipid oxidation compared to untreated controls [32,40,41,50,51]. For dairy products, CPAW pretreatment preserves vitamins and antioxidants while reducing spoilage microbes (Table 2). These characteristics make CPAW particularly suitable for fresh-cut and ready-to-eat applications, including organic products where synthetic preservatives are restricted. However, quality responses to CPAW are not uniformly beneficial. While some studies report improved firmness, color retention, and preservation of antioxidants, others have observed pigment degradation or reduced antioxidant activity, particularly under more intensive treatment conditions. These apparently contradictory findings can be explained partly by the dual role of RONS. At moderate concentrations, oxidative stress may suppress spoilage organisms and enzymes or stimulate protective responses, whereas excessive RONS exposure can oxidize phenolic compounds, pigments, lipids, and vitamins. The food matrix further modifies this response because compounds with high antioxidant capacity may initially scavenge RONS and protect quality, but they may also reduce the antimicrobial effectiveness of CPAW. Thus, increasing CPAW treatment intensity does not necessarily produce a proportional improvement in overall food quality, and an antimicrobial–quality trade-off should be considered when defining optimal processing conditions. In evaluating these trade-offs, assessment of nutritional and sensory quality should be carried out in addition to microbial inactivation. Relevant measurements include changes in vitamin content, phenolic compounds, lipid oxidation, color, texture, flavor, and consumer acceptability. Enhanced microbial control should not be assumed to indicate equivalent preservation of nutritional or sensory quality since these responses depend on the food matrix and treatment conditions.

#### 2.4.4. Modification of Functional Ingredients

Research has increasingly examined CPAW as a tool for modifying functional ingredients. Treatment can improve water absorption, solubility, gel strength, and thermal stability of starches, proteins, and polysaccharides with potential benefits for clean-label formulations [13,16,49,52]. These effects are attributed to gentle oxidation of hydroxyl groups and polymer depolymerization or cross-linking. These functional modifications represent an emerging research direction with considerable potential for food formulation and ingredient development.

#### 2.4.5. Biofilm Control and Equipment Sanitation

In food-processing facilities, CPAW has potential applications in microbial control on food-contact surfaces and processing equipment such as cutting boards, conveyor belts, and dairy-processing systems [10]. Its antimicrobial activity is related to plasma-generated reactive species and the physicochemical conditions of the activated water. However, performance depends on biofilm composition, surface material, organic load, treatment duration, and the accessibility of embedded microorganisms.

Plasma-based water treatment also provides a related opportunity for managing process-water quality and supporting water-reuse strategies. Nevertheless, the direct purification of contaminated water and the subsequent use of CPAW as a sanitizing medium represent distinct operations. A reduction in microbial contamination or chemical pollutants in water does not, by itself, establish that the treated water is suitable for direct food contact. Accordingly, water quality, potential reaction products, residual reactive species, and compatibility with equipment materials must be evaluated for the intended application [53]. For cleaning-in-place systems, CPAW should be assessed against established cleaning and sanitization requirements, including removal of food residues and validated microbial control, rather than assuming that antimicrobial activity alone ensures complete cleaning [54]. Although CPAW shows potential for equipment sanitation and cleaning-in-place applications, further validation is needed under actual industrial operating conditions before its routine commercial implementation can be established.

#### 2.4.6. Growth-Promoting and Signaling Effects in Agriculture

In addition to its bactericidal activity, CPAW has been reported to stimulate growth and physiological responses in agricultural and postharvest systems, primarily through reactive nitrogen species and calcium-mediated signaling pathways. Nitrate and nitrite present in CPAW serve as bioavailable nitrogen sources, while low levels of oxidative stress can act as signaling stimuli rather than causing cellular damage. Current evidence suggests that CPAW induces transient changes in cytosolic Ca^2+^ levels, thereby initiating calcium-mediated signaling cascades involved in cellular regulation [10,29]. These signaling cascades activate key metabolic enzymes and enhance nutrient uptake, thereby promoting seed germination, root development, and overall crop vigor. Therefore, CPAW exhibits dual functionality by controlling phytopathogens while regulating physiological processes, with these effects strongly influenced by RONS concentration and exposure duration.

**Table 2:** Summary of CPAW applications across food categories and observed outcomes.

Food Matrix	Target Microorganism/Outcome	Plasma Source/Configuration	Power/Voltage	pH/ORP	Key RONS Concentration	Application and Storage Conditions	Main Outcome	References
Fresh Produce (lettuce, strawberries, blueberries)	*E. coli*, *Salmonella*, *L. monocytogenes*, *B. cereus*, yeasts, and molds	DBD, surface DBD, diffusor (DBDD), or pin-to-plate using ambient air or He/O_2_	12–36 W or 6–11 kV peak voltage across system designs	pH drop to 2.4–3.0; elevated ORP up to >540–576 mV	Superoxide and peroxynitrite	Washing or immersion for 0.5–5 min; storage at 3–5 °C or 25 °C	1.0–7.5 log CFU/g pathogen reduction; extended shelf life without chemical residues	[32,39,40,41,55]
Meat and Poultry	*E. coli*, *S. aureus*, *Salmonella*, *Vibrio*, *psychrotrophs*, yeasts, and molds	DBD, plasma jet, transient spark, or pin-to-plate with ambient air, Ar, or O_2_	12–750 W or 6–18 kV	Acidic pH (drop to 2.0–3.5) and elevated ORP	Aqueous, peroxynitrite and free radicals	Washing, spraying, or immersion (1–15 min); stored refrigerated at 3–5 °C	0.7 to 6.0 log CFU reductions; extended shelf life and potential meat curing	[22,42,43,56,57]
Seafood	*Vibrio cholerae*, *Vibrio parahaemolyticus*, *E. coli* and *Staphylococcus aureus*	Flow-through jet, SDBD, pulsed corona, pin-to-liquid, and piezoelectric direct discharge	10–30 kV, high-voltage DBD at 160 kV, or 40–750 W	Strong acidification (pH drops to 2.7–3.6) and oxidation-reduction potential between 246 and 550 mV	Peroxynitrous acid, and nitrous acid	Washing fish filets and extends product shelf life under refrigerated conditions at 3–4 °C	1.3 to 4.4 log CFU reductions in pathogens; extended freshness by 4–8 days	[44,45,46,58,59]
Dairy (milk, cheese surfaces)	*L. monocytogenes*, *L. innocua*, *S. aureus*, *E. coli*, *P. aeruginosa*, coliforms	Dielectric barrier discharge (DBD), plasma bubble reactors, SDBD, and corona units	16–36 W or 2–45 kV (up to 80 kV)	Acidic pH (2.4–3.0 in pure water; ~6.59 buffered in milk); ORP 540–589 mV	Aqueous, peroxynitrite and free radicals	Liquid contact, soaking, or CIP washing at 20–75 °C for 1 to 20 min Refrigerated holding at 4 °C	1.2 to 8.0 log microbial inactivation; milk fat oxidation limits high doses	[53,60,61]
Juices and Beverages	*E. coli*, *S. cerevisiae*, *A. acidoterrestris*, *total plate count*, and yeasts/molds	Dielectric barrier discharge (DBD), bubble DBD, atmospheric jet, corona, and transient spark	16–45 kV (up to 80 kV) or 16–650 W	Slight pH decrease (0.1–0.3 units) in buffered juices; ORP rises with applied voltage	Superoxide and transient hydroxyl radicals	Direct bubbling, batch electrospray, or membrane filtration cleaning, refrigerated holding at 4–10 °C for 6 to 26 days	1.0 to 6.0 log microbial reduction, preserved polyphenols, and shelf-life extension	[62,63]

### 2.5. Comparative Evaluation of CPAW and Other Non-Thermal Sanitization Technologies

Table 3 compares CPAW with selected non-thermal sanitization technologies used in food processing, highlighting their mechanisms of action, target microorganisms, effects on food quality, environmental impacts, and key limitations. CPAW inactivates microorganisms through the combined action of RONS, reduced pH, and elevated oxidation–reduction potential, enabling effective control of bacteria, yeasts, molds, and spores [7,15,64]. Because this action occurs under non-thermal conditions, nutrient content, color, and texture are better preserved than with heat-based alternatives. CPAW may also offer environmental benefits through reduced water consumption, lower chemical usage, and relatively low energy requirements. The antimicrobial performance of CPAW can be substantial even at relatively short treatment times. Hadinoto et al. [56] achieved 3.99 ± 0.13 and 5.90 ± 0.24 log_10_ reductions in *Escherichia coli* and *Salmonella typhimurium*, respectively, within 300 and 240 s, while an energy efficiency of 10.1 ± 0.1 g kW^−1^ h^−1^ was obtained for RONS generation. Nonetheless, CPAW implementation is limited by the need for plasma-generation equipment and the short-lived stability of RONS during storage. The comparatively high microbial reductions observed by Hadinoto et al. [56] should not be interpreted as evidence that CPAW consistently achieves greater efficacy than the lower reductions reported in other food systems. Direct comparison is complicated by differences in experimental design, particularly whether microorganisms were treated in suspension or on actual food surfaces, the initial inoculum level, and the composition of the treatment matrix. Microbial cells in simple aqueous systems are generally more accessible to reactive species than cells attached to irregular or nutrient-rich food surfaces, where organic matter may quench RONS and reduce their antimicrobial availability [65]. Therefore, reported log reductions should be interpreted within the context of the experimental matrix rather than as universally comparable indicators of CPAW performance.

Other non-thermal technologies differ in antimicrobial mechanisms and practical constraints. UV-C irradiation relies on thymine dimer formation to damage microbial DNA and is efficient for surface decontamination, though its penetration is limited and shadowing reduces effectiveness on irregularly shaped produce [66]. Under appropriate irradiation conditions, UV-C can achieve microbial reductions in the range of approximately 2–5 log_10_, although the effectiveness depends strongly on UV dose, microorganism, and the optical properties and geometry of the treated matrix [66,67,68]. Ozone, whether used in aqueous or gaseous form, offers strong oxidative action against a broad spectrum of microbes but may affect food aroma or flavor if overdosed, and its corrosive nature requires strict safety protocols [69]. Ozonation has similarly been associated with several-log reductions in foodborne microorganisms, with the extent of inactivation governed by ozone concentration, exposure time, microorganism, and organic load of the treatment medium [70]. Pulsed Electric Fields (PEF) provide excellent quality retention for liquid foods via electroporation but are limited to conductive liquid matrices [71,72]. Depending on the applied electric-field strength and treatment conditions, PEF can achieve approximately 1–5 log_10_ reductions in susceptible vegetative microorganisms, while requiring relatively short treatment times because microbial inactivation occurs through electroporation of the cell membrane [71]. High-pressure processing (HPP) preserves fresh-like attributes while disrupting microbial membranes; however, high equipment costs and reduced efficiency in porous foods remain notable limitations [73,74]. HPP treatments commonly achieve microbial reductions of approximately 3–6 log_10_ for vegetative microorganisms, although the magnitude of inactivation depends on pressure, holding time, temperature, microorganism, and food matrix [75]. Ultrasound combined with mild heat inactivates microorganisms through cavitation-induced shear forces, yet its effectiveness is often lower when applied alone and it can induce texture changes depending on the product [76]. Ultrasound alone may provide relatively modest microbial reductions, often below 2 log_10_, whereas combining ultrasound with mild heat or other antimicrobial hurdles can increase the reduction to approximately 2–5 log_10_, depending on the treatment intensity and food matrix [77,78]. 

Generally, compared with these non-thermal sanitization technologies, available evidence suggests CPAW may offer a favorable balance between antimicrobial efficacy and product quality preservation, although its industrial advantages depend on process conditions and application requirements. From an energy-efficiency perspective, recent evidence further suggests the potential of PAW systems to achieve substantial microbial inactivation with low electrical energy input. A dielectric barrier discharge microbubble system achieved energy efficiencies of 5.463–8.554 log_10_ kWh^−1^ against *Candida albicans*, *Staphylococcus aureus*, and *E. coli*, with electrical energy inputs of only 0.000146–0.000292 kWh under the tested conditions [47]. However, challenges related to the stability of reactive species, equipment requirements, and scalability must be addressed to support broader industrial adoption. Because the studies available for CPAW, UV-C, ozone, PEF, HPP, and ultrasound differ substantially in target microorganisms, inoculum levels, food matrices, treatment intensity, exposure time, and outcome measurement, the numerical reductions reported below should not be interpreted as a head-to-head ranking of technologies. Quantitative comparisons are most meaningful only when treatment conditions and biological systems are comparable; therefore, the values are presented primarily to indicate experimentally reported performance ranges and should be interpreted together with their respective treatment conditions.

The effectiveness of CPAW can be further enhanced when integrated with complementary preservation strategies, as illustrated in Table 3. Combining CPAW with moderate heat, ultrasound, essential oils, or organic acids often produces synergistic antimicrobial outcomes. CPAW can enhance the permeability of microbial membranes, thereby improving the penetration and activity of natural antimicrobials [64,79]. Likewise, using CPAW as a pre-treatment step can reduce the thermal load required for subsequent heat processing, helping maintain heat-sensitive nutrients, textures, and sensory properties. Such combinations can therefore improve the overall process efficiency by achieving greater microbial reductions without proportionally increasing treatment intensity. These synergistic interactions support the integration of CPAW into multi-hurdle preservation systems tailored for minimally processed foods.

An additional consideration in comparing CPAW with conventional chemical sanitizers is the potential for microbial adaptation following repeated sublethal exposure. CPAW acts through multiple oxidative and nitrosative stresses affecting membranes, proteins, and nucleic acids, which may reduce the likelihood of resistance arising through a single target-specific mechanism [80,81]. However, this should not be interpreted as evidence that CPAW is incapable of selecting tolerant populations. Microorganisms possess oxidative-stress defense and repair systems, and evidence concerning adaptation following repeated sublethal exposure to plasma-derived RONS remains limited and system-dependent [32]. Consequently, the potential for CPAW-induced tolerance, cross-protection, or changes in antimicrobial susceptibility after repeated sublethal treatment requires systematic investigation before a lower antimicrobial-resistance risk than conventional sanitizers can be established.

**Table 3:** Comparison of CPAW with selected non-thermal and hybrid sanitization technologies in food processing.

Technology	Mechanism of Action	Target Microorganisms	Effect on Food Quality	Environmental Impact	Limitations	References
CPAW (Cold Plasma Activated Water)	Reactive oxygen and nitrogen species (RONS), low pH, high ORP	Bacteria, yeasts, molds, spores	Minimal nutrient loss, good retention of texture and color	Potential for reduced chemical and water demand	Requires plasma generator; unstable RONS with time	[7,39,82]
UV-C Irradiation	DNA damage via thymine dimer formation	Surface bacteria and some viruses	No heat impact; possible discoloration of sensitive produce	Minimal energy; no chemical waste	Limited penetration; shadowing effect	[66,67,68]
Ozone (aqueous/gaseous)	Strong oxidation of cell walls and proteins	Broad-spectrum bacteria, viruses, fungi	May affect flavor and aroma if overdosed	Decomposes into oxygen; residue-free	Corrosive; strict safety protocols	[69,70]
Pulsed Electric Fields (PEF)	Electroporation of microbial membranes	Primarily bacteria in liquids	Excellent retention of flavor and color in juices	Moderate power demand; no toxic chemicals	Only suitable for conductive liquids	[72,83]
High Pressure Processing (HPP)	Cell membrane disruption by pressure	Bacteria, yeasts, molds	Retains fresh attributes; minor protein denaturation	High initial equipment cost; water recycling needed	Not suitable for porous foods	[73,74,75]
Ultrasound	Cavitation-induced shear and localized heating	Spoilage microbes, enzymes	Can modify texture; moderate heat effect	Low chemical input	Limited efficacy when used alone	[76,77]
CPAW + Mild Heat	Synergistic inactivation via membrane disruption, ROS/RNS, mitochondrial damage	*E. coli*, *S. cerevisiae*	Maintains sensory quality	Reduced chemical dependency; energy-efficient	Species instability; scale-up challenges	[84,85,86]
CPAW + Ultrasound	Cavitation, enhanced ROS/RNS penetration, biofilm disruption	*E. coli*, *A. veronii*, biofilms	Biofilm inhibition, shelf-life	Low chemical input; minimal secondary waste	Process optimization, species stability, scale-up, penetration	[57,87]
CPAW + Organic Acids/Naturals	Synergistic RONS/acid action, enzyme inhibition, metabolic disruption	*S. aureus*, *E. coli*, fungi	Delays spoilage, preserves quality	Reduced synthetic sanitizer demand; low waste generation	RONS/acid stability, process optimization, scale-up challenges	[80,81,88]
CPAW + In-package Plasma	Synergistic RONS action, in-package decontamination, packaging modification	Mixed foods	Enhanced storage stability	Reduced sanitizer use; compatible with sustainable packaging	Process optimization, quality changes, scale-up challenges	[89,90,91]

### 2.6. Sustainability, Safety, and Regulatory Considerations of CPAW in Food Processing

As research interest in CPAW grows, its alignment with sustainability goals, food safety regulations, and industry standards has come to deserve serious attention. These considerations are critical for successful industrial implementation, market acceptance, and the long-term integration of CPAW into commercial food-processing systems.

#### 2.6.1. Environmental Sustainability and Green Processing

From an environmental standpoint, CPAW has the potential to reduce dependence on conventional chemical sanitizers and energy-intensive thermal preservation processes. Depending on the reactor configuration and intended application, CPAW may reduce the need for externally added chemical sanitizers. However, its overall environmental performance depends on reactor configuration, energy efficiency, feed gas requirements, water consumption and reuse, equipment manufacture, treatment conditions, and the formation or disposal of residual reaction products [25].

The environmental performance of CPAW cannot yet be generalized because it depends on reactor efficiency, gas supply, water volume, treatment intensity, equipment lifetime, and the conventional process being displaced. Quantitative energy measurements are available for some PAW reactor configurations. Ferreira et al. [47] reported electrical energy inputs of 0.000146–0.000292 kWh for microbial inactivation using a dielectric barrier discharge microbubble system under the specific experimental conditions evaluated. However, such reactor-level measurements are not equivalent to a full life-cycle assessment. Comparative data on water consumption, chemical-sanitizer displacement, wastewater treatment requirements, equipment manufacture, and full-scale energy demand remain sparse. Consequently, claims of lower carbon footprint or overall environmental superiority should presently be regarded as promising but not conclusively established. The relatively low electrical energy inputs reported for some CPAW systems indicate potential for energy-efficient operation; however, whether this translates into a lower overall carbon footprint than conventional preservation or sanitation processes requires comparative life-cycle assessment [12,25].

Experience from plasma-based water purification provides useful engineering information for evaluating CPAW sustainability, particularly regarding reactor energy consumption, gas–liquid mass transfer, water throughput, and process integration [25,92]. However, environmental benefits cannot be inferred from plasma activation alone. A meaningful comparison should consider electrical energy per unit volume treated, the required water quality, gas consumption, electrode durability, potential transformation products, and any subsequent treatment or disposal requirements [25,93]. In food-processing applications, these factors should be assessed alongside the antimicrobial and quality outcomes achieved by the generated CPAW.

#### 2.6.2. Food Safety and Toxicological Considerations

Although CPAW has been widely shown to effectively inactivate microorganisms, the safety of CPAW-treated foods remains a major consideration. One major concern is the stability and residual RONS present in the solution after plasma activation. Many short-lived reactive species decay rapidly after treatment; however, the identity, concentration, and toxicological significance of residual species and matrix-specific reaction products require further characterization [10].

Available toxicological studies have not identified major adverse effects under the specific experimental conditions investigated [7,10]. However, the evidence base remains limited, particularly regarding chronic exposure, genotoxicity, allergenicity, and the formation of matrix-specific reaction products. Consequently, current findings should not be interpreted as establishing comprehensive long-term safety. Furthermore, microbial inactivation data alone are insufficient to establish the toxicological safety of CPAW-treated foods. Future safety assessments should therefore quantify residual RONS at the point of consumption, identify secondary reaction products formed through interactions with lipids, proteins, phenolics, and other food constituents, establish their toxicological relevance, and evaluate repeated or chronic exposure under realistic dietary conditions.

Where CPAW is generated from water that has previously undergone plasma-based purification, the assessment must distinguish removal of the original contaminants from the possible formation or persistence of plasma-induced transformation products. The presence of antimicrobial reactive species should not be treated as evidence of potable or food-grade water quality. Both the starting water and the final activated water require characterization appropriate to their intended use, particularly when direct contact with food is anticipated.

#### 2.6.3. Regulatory Landscape

The regulatory assessment of CPAW remains a challenge for its application in food processing. Although established food-safety frameworks exist in major jurisdictions, including the United States, European Union, China, and Japan, these do not necessarily constitute specific authorization for CPAW. In the United States, food-processing applications may be subject to the Federal Food, Drug, and Cosmetic Act and relevant provisions of Title 21 of the Code of Federal Regulations, depending on the intended use and regulatory classification [94]. In the European Union, Regulation (EC) No. 178/2002 establishes general food-law requirements, while Regulation (EC) No. 852/2004 addresses food hygiene; Regulation (EU) No. 528/2012 may also be relevant where a product falls within the scope of biocidal-product legislation [95,96,97].

Regulatory requirements may vary depending on the intended application, food-contact conditions, residual reactive species, and potential reaction products. Currently, the literature reviewed provides insufficient authoritative evidence to establish CPAW-specific approval status across these jurisdictions. Therefore, regulatory assessment should be jurisdiction- and application-specific, considering antimicrobial efficacy, chemical characterization, toxicological safety, and compliance with relevant food-safety requirements. Further engagement with regulatory authorities is needed to clarify approval pathways for commercial applications. Importantly, for every CPAW application, compliance with general food-safety legislation should not be understood as specific regulatory authorization.

#### 2.6.4. Consumer Perception and Labeling

As consumer demand for minimally processed and low-chemical foods increases, CPAW may become an appealing preservation technology. However, the term “plasma” may generate consumer concern because of limited public familiarity with the technology. Clear communication strategies and transparent labeling may help build consumer trust [98].

Educating consumers about the water-based nature and environmental benefits of CPAW may help reduce skepticism and improve acceptance. Consumer studies suggest that acceptance improves significantly when the safety and sustainability benefits of CPAW are clearly communicated [99].

### 2.7. Current Challenges, Research Gaps, and Future Directions

Despite the growing interest in CPAW for food-processing applications, several technical and translational challenges remain. These limitations must be addressed to enable effective commercialization of CPAW, optimize its performance across food matrices, and improve regulatory and consumer acceptance.

In considering these research gaps, it is important to distinguish between established observations, proposed mechanisms, and research hypotheses. Established evidence includes experimentally measured changes such as CPAW acidification, elevated oxidation–reduction potential, formation of long-lived RONS, and microbial inactivation under defined experimental conditions. In contrast, mechanistic interpretations—including the relative contributions and interactions of individual RONS, their diffusion and reactions within complex food matrices, and the molecular pathways responsible for some quality changes—remain partly inferred and condition-dependent. Similarly, anticipated benefits associated with industrial-scale implementation, process integration, long-term safety, and environmental performance should be regarded as areas requiring further validation rather than established advantages where supporting pilot-scale, toxicological, or life-cycle evidence remains limited. The following research priorities are therefore discussed with these different levels of evidence in mind.

#### 2.7.1. Mechanistic Uncertainties and Optimization

Understanding how CPAW interacts with different food constituents at the molecular level is one of the most pressing research needs. Although the antimicrobial and redox effects of RONS are generally understood, their precise behavior in complex food systems remains unclear. In particular, the formation of secondary by-products and the interactions of RONS with proteins, lipids, and polyphenols require further investigation [100]. In addition, the stability and effective lifetime of RONS in aqueous and food matrices require further characterization, especially under varying pH, ionic strength, and temperature conditions.

Advanced analytical tools such as real-time electron paramagnetic resonance (EPR) spectroscopy, ultra-high-performance liquid chromatography–mass spectrometry (UHPLC-MS), and nuclear magnetic resonance (NMR) can provide deeper insight into the molecular mechanisms of CPAW. These tools may also support the development of predictive models for optimizing CPAW treatment across different food categories.

#### 2.7.2. Standardization and Scalability

Within the scope of the literature reviewed, there are few, if any, standardized protocols for CPAW generation, treatment conditions, and performance evaluation. Plasma source configuration, gas composition, discharge voltage, and treatment duration all significantly influence RONS generation and treatment outcomes. These variations reduce reproducibility and make cross-study comparisons and industrial scale-up more challenging [101]. A further problem is that many studies report nominal treatment time without reporting the actual chemical dose delivered by CPAW. Two treatments described as 10 min CPAW exposure may differ substantially in H_2_O_2_, NO_2_^−^, NO_3_^−^, ORP, and short-lived species because of differences in reactor design and operating conditions. Consequently, treatment time cannot presently serve as a universal basis for comparing CPAW efficacy. The development of standardized dose metrics linking electrical energy input and measurable RONS concentrations to microbial or enzymatic responses is therefore necessary for reproducible process design. The broader plasma-based water-treatment literature offers established reactor concepts relevant to CPAW scale-up, including surface discharges, gliding arc systems, submerged electrodes, and bubble-assisted configurations. However, reactor performance should be evaluated against the intended treatment objective. Energy efficiency expressed as contaminant removal per unit energy in wastewater treatment is not directly interchangeable with reactive-species generation efficiency or microbial inactivation performance in food-processing applications. Future studies should report reactor configuration, treated-water volume, electrical energy input, gas flow, initial water composition, reactive-species concentrations, and treatment performance using clearly defined and comparable metrics.

Standardized CPAW processing procedures and validation matrices must be developed in order to facilitate commercialization. Although most CPAW studies remain laboratory-scale, initial pilot-scale and continuous systems have been reported. Andrasch et al. [102] demonstrated plasma-processed water generation in a pilot-plant salad-rinsing system, while subsequent pilot-scale PAW treatment has been evaluated for products including romaine lettuce and steamed rice cakes [103,104]. More recently, continuous PAW generation and application systems integrating reactive-species production with continuous fresh-produce washing have also been developed [104]. These studies demonstrate the technical feasibility of scale-up, but evidence at sustained commercially relevant throughput remains limited. Further pilot and industrial validation should therefore focus on continuous reactive-species control, treatment uniformity, energy and water demand, equipment compatibility, throughput, and techno-economic performance under realistic food-processing conditions.

#### 2.7.3. Interaction with Food Matrix and Quality Preservation

CPAW has shown potential in reducing microbes and inactivating enzymes; however, its effects on sensory, nutritional, and structural attributes remain a major concern. Inconsistent effects on texture, color, and antioxidant activity have been reported depending on the food matrix and CPAW treatment conditions [105].

Comprehensive quality evaluations involving physicochemical, sensory, and shelf-life analyses are needed to assess the overall impact of CPAW on food integrity. Future studies should determine whether combining CPAW with mild preservation strategies such as vacuum packaging, edible coatings, or pulsed light produces synergistic effects that improve antimicrobial efficacy while minimizing undesirable quality changes. 

#### 2.7.4. Regulatory Approval and Public Perception

As discussed previously, the standard for regulating CPAW has yet to be established. The lack of harmonized international regulations, combined with limited long-term safety studies, complicates product approval and commercialization. More targeted risk assessments and toxicological studies of CPAW-treated foods under realistic use conditions are also needed.

From the consumer’s perspective, misguided perceptions toward plasma technology and its potential link to radiation or “artificial” treatment should be addressed through evidence-based education and communication strategies. Consumer attitudes and market acceptability research can help in directing labeling regulations and communication strategies presenting the available safety evidence, remaining uncertainties, and potential environmental implications of CPAW. 

#### 2.7.5. Research Outlook

Future CPAW research will benefit from stronger interdisciplinary collaboration among food technologists, plasma physicists, materials scientists, and toxicologists. Advancing plasma engineering to tailor the composition, concentration, and lifetime of reactive oxygen and nitrogen species will be essential for achieving targeted inactivation of specific spoilage organisms, pathogens, or enzymes. Parallel efforts should focus on designing robust, food-grade CPAW generation and delivery systems that ensure consistent performance, treatment uniformity, and scalability for commercial processing environments. Also, future studies should perform comparative life-cycle assessments between CPAW and chlorine-based sanitization to quantify environmental benefits.

Additional opportunities lie in integrating CPAW with smart technologies, such as biosensors or inline monitoring tools, to enable real-time assessment of microbial load or product quality during processing. Emerging research should also explore how CPAW influences bioactive compounds, structural components, and functional attributes of foods, with attention to both beneficial modifications and potential safety considerations. Collectively, these research directions will accelerate the transition of CPAW from laboratory research to reliable industrial-scale food processing applications.

## 3. Conclusions

Cold plasma-activated water (CPAW) is a promising non-thermal method for microbial control, enzyme modulation, and modification of food components. The effectiveness of CPAW is due to the changes in reactive species and physicochemical properties generated during plasma activation. However, treatment outcomes depend on the processing conditions, composition of the activated water, and nature of the treated food. Although experimental studies have shown potential benefits, these findings should not be interpreted as evidence of established industrial readiness or uniform performance across food systems.

This review has examined the fundamental principles, physicochemical properties, and mechanisms of action of CPAW, as well as its applications in food preservation, structural modification, and quality retention. It also highlights the role of advanced characterization tools and predictive modeling in improving process control, reproducibility, and treatment optimization. Recent studies demonstrate the effectiveness of CPAW across a wide range of food products, including fruits, vegetables, juices, dairy products, and cereals, where it has contributed to improved shelf stability, safety, and quality retention.

There is a need to address several research gaps prior to the widespread implementation of CPAW in food processing. These include the development of reproducible treatment procedures and physicochemical dose descriptors, clarification of the combined and individual contributions of reactive species, and justification of antimicrobial performance under industrially relevant conditions. Further research is also required to determine or establish the efficacy of CPAW on nutritional and sensory quality, characterize potential reaction products and long-term toxicological risks, and determine application-specific regulatory requirements. Coordinated experimental, engineering, and safety studies will thereby be essential towards addressing these limitations for evaluating the practical suitability of CPAW as a food-processing technology.

The development of CPAW should also be understood within the broader field of plasma-based water treatment, which has established important principles concerning plasma–liquid interactions, contaminant degradation, microbial inactivation, and reactor engineering. Surface discharges, gliding arc systems, submerged discharges, and plasma generated within gas bubbles provide different approaches for transferring plasma-induced reactivity into water. Nevertheless, the requirements for water purification and CPAW production are not identical. Translating these reactor concepts into food-processing applications requires control of the resulting aqueous chemistry, verification of water quality, and assessment of food-specific safety and performance.

The principal barrier to CPAW commercialization is therefore no longer demonstrating antimicrobial activity, but establishing reproducible relationships between plasma operating conditions, measurable chemical dose, food–matrix interactions, microbial or enzymatic responses, product quality, safety, and process economics. Future research should shift from treatment-time-based reporting toward standardized physicochemical dose descriptors and pilot-scale validation under realistic processing conditions. Until comparable life-cycle, toxicological, and techno-economic data become available, claims regarding the superiority and sustainability of CPAW over established sanitization technologies should remain application-specific.

Future research should now move beyond demonstrations of antimicrobial efficacy toward four interrelated translational priorities. First, reproducibility requires standardized reporting of plasma source and geometry, feed gas, discharge power, activation time, water composition and volume, pH, ORP, conductivity, key RONS concentrations, storage interval, treatment-to-product ratio, and application conditions. Second, industrial scale-up requires pilot-scale validation of continuous CPAW generation and delivery, reactive-species stability, process control, energy and water demand, equipment compatibility, and techno-economic performance under realistic food-processing conditions. Third, safety validation should establish residual RONS at consumption, identify matrix-specific secondary reaction products, and evaluate chronic toxicity, genotoxicity, allergenicity, and realistic dietary exposure. Finally, regulatory translation will require standardized efficacy and safety protocols, validated analytical methods, clearly defined intended uses, and jurisdiction-specific datasets suitable for regulatory assessment. Addressing these priorities is essential before the promising laboratory-scale performance of CPAW can be translated into reproducible, safe, economically viable, and regulatorily acceptable food-processing applications.

## Data Availability

No new data were created or analyzed in this study. Data sharing is not applicable to this article.

## References

[1] Zhang Z.H., Wang L.H., Zeng X.A., Han Z., Brennan C.S. (2019). Non-thermal technologies and its current and future application in the food industry: A review. Int. J. Food Sci. Technol..

[2] Han Q.Y., Wen X., Gao J.Y., Zhong C.S., Ni Y.Y. (2023). Application of plasma-activated water in the food industry: A review of recent research developments. Food Chem..

[3] Wu R., Feng J., Yue M., Liu D., Ding T. (2023). Overuse of food-grade disinfectants threatens a global spread of antimicrobial-resistant bacteria. Crit. Rev. Food Sci. Nutr..

[4] Shanker M.A., Khanashyam A.C., Pandiselvam R., Joshi T.J., Thomas P.E., Zhang Y., Rustagi S., Bharti S., Thirumdas R., Kumar M. (2023). Implications of cold plasma and plasma activated water on food texture—A review. Food Control.

[5] Vedaei S., Dara A. (2025). Surveying the utilization of cold plasma and plasma-activated water on food pigments, bioactive compounds, enzymes, vitamins, fatty acid, and essential oils: Considerations, mechanisms, and future trends. Food Chem. X.

[6] Nasiru M.M., Frimpong E.B., Muhammad U., Qian J., Mustapha A.T., Yan W., Zhuang H., Zhang J. (2021). Dielectric barrier discharge cold atmospheric plasma: Influence of processing parameters on microbial inactivation in meat and meat products. Compr. Rev. Food Sci. Food Saf..

[7] Niedźwiedź I., Waśko A., Pawłat J., Polak-Berecka M. (2019). The state of research on antimicrobial activity of cold plasma. Pol. J. Microbiol..

[8] Zeghioud H., Nguyen-Tri P., Khezami L., Amrane A., Assadi A. (2020). Review on discharge Plasma for water treatment: Mechanism, reactor geometries, active species and combined processes. J. Water Process Eng..

[9] Bruggeman P.J., Kushner M.J., Locke B.R., Gardeniers J.G.E., Graham W.G., Graves D.B., Hofman-Caris R.C.H.M., Maric D., Reid J.P., Ceriani E. (2016). Plasma–liquid interactions: A review and roadmap. Plasma Sources Sci. Technol..

[10] Gao Y., Francis K., Zhang X. (2022). Review on formation of cold plasma activated water (PAW) and the applications in food and agriculture. Food Res. Int..

[11] Misra N.N., Yadav B., Roopesh M.S., Jo C. (2019). Cold plasma for effective fungal and mycotoxin control in foods: Mechanisms, inactivation effects, and applications. Compr. Rev. Food Sci. Food Saf..

[12] Thirumdas R., Kothakota A., Annapure U., Siliveru K., Blundell R., Gatt R., Valdramidis V.P. (2018). Plasma activated water (PAW): Chemistry, physico-chemical properties, applications in food and agriculture. Trends Food Sci. Technol..

[13] Sasikumar R., Mangang I.B., Kaviarasu G., Kaushik R., Mansingh P., Tomer V., Jaiswal A.K. (2025). A comprehensive review on cold plasma applications in the food industry. Sustain. Food Technol..

[14] Li B., Peng L., Cao Y., Liu S., Zhu Y., Dou J., Yang Z., Zhou C. (2024). Insights into Cold Plasma Treatment on the Cereal and Legume Proteins Modification: Principle, Mechanism, and Application. Foods.

[15] Xu H., Zhu Y., Du M., Wang Y., Ju S., Ma R., Jiao Z. (2021). Subcellular mechanism of microbial inactivation during water disinfection by cold atmospheric-pressure plasma. Water Res..

[16] Tolouie H., Mohammadifar M.A., Ghomi H., Hashemi M. (2018). Cold atmospheric plasma manipulation of proteins in food systems. Crit. Rev. Food Sci. Nutr..

[17] Kovačević V., Sretenović G., Obradović B., Kuraica M. (2022). Low-temperature plasmas in contact with liquids—A review of recent progress and challenges. J. Phys. D Appl. Phys..

[18] Hong J., Zhang T., Zhou R., Ostikov K., Rezaeimotlagh A., Cullen P.J. (2021). Plasma bubbles: A route to sustainable chemistry. AAPPS Bull..

[19] Schneider M., Rataj R., Bláha L., Kolb J. (2022). Experimental review of different plasma technologies for the degradation of cylindrospermopsin as model water pollutant. Chem. Eng. J..

[20] Miranda F., Tavares V., Gomes M., Neto N.F.A., Chiappim W., Petraconi G., Pessoa R.S., Koga-Ito C.Y. (2023). Physicochemical Characteristics and Antimicrobial Efficacy of Plasma-Activated Water Produced by an Air-Operated Coaxial Dielectric Barrier Discharge Plasma. Water.

[21] Simon S., Salgado B., Hasan M., Sivertsvik M., Fernandez E., Walsh J. (2021). Influence of Potable Water Origin on the Physicochemical and Antimicrobial Properties of Plasma Activated Water. Plasma Chem. Plasma Process..

[22] Agus R., Pipoz L., Avino F., Lavrikova A., Myers B., Furno I. (2024). Plasma-activated water retains antimicrobial properties against Escherichia coli after 72 h of storage. Plasma Phys. Control. Fusion.

[23] Song Z.Q., Jiang Y., Chen C., Ding C.J., Chen H. (2024). Effect of Plasma-Activated Water on the Cellulase-Producing Strain *Aspergillus niger* A32. J. Fungi.

[24] Yang X., Zhang C., Li Q., Cheng J.H. (2023). Physicochemical properties of plasma-activated water and its control effects on the quality of strawberries. Molecules.

[25] Bexeitova K., Zhantikeyev U., Kanapiya M., Toktar M., Lee J., Azat S. (2026). Cold plasma technologies for sustainable water treatment: A state-of-the-art review. Rev. Chem. Eng..

[26] Wong K.S., Chew N.S.L., Low M., Tan M.K. (2023). Plasma-activated water: Physicochemical properties, generation techniques, and applications. Processes.

[27] Rotondo P., Aceto D., Ambrico M., Stellacci A.M., Faretra F., Angelini R.M.D.M., Ambrico P.F. (2025). Physicochemical properties of plasma-activated water and associated antimicrobial activity against fungi and bacteria. Sci. Rep..

[28] Cai Z.C., Wang J.M., Wang Y.Y., Sang X., Zeng L., Deng W., Zhang J. (2023). Effect of different process conditions on the physicochemical and antimicrobial properties of plasma-activated water. Plasma Sci. Technol..

[29] Ali N., Dong Y. (2026). Global meta-analysis of plasma-activated water for improving crop establishment, productivity, and health. Front Plant Sci..

[30] Kooshki S., Pareek P., Janda M., Machala Z. (2024). Selective reactive oxygen and nitrogen species production in plasma-activated water via dielectric barrier discharge reactor: An innovative method for tuning and its impact on dye degradation. J. Water Process Eng..

[31] Cingesar I.K., Stulić V., Markić F., Muratović S., Kurek M., Herceg Z., Maltar-Strmečki N., Pavičić T.V. (2025). Aging Stability and Radical Activity of Plasma-Activated Water Treated in Liquid- and Gas-Phase Reactors. Molecules.

[32] Triantaphyllidou I.E., Aggelopoulos C. (2024). Insights on bacteria inactivation in water by cold plasma: Effect of water matrix and pulsed plasmas waveform on physicochemical water properties, species formation and inactivation efficiency of *Escherichia coli*. Environ. Res..

[33] Lunder M., Dahle S., Poljšak B., Fink R. (2026). The power of neutralization: The critical step for the accurate antimicrobial potential of plasma-activated water. Front Microbiol..

[34] Surowsky B., Fischer A., Schlueter O., Knorr D. (2013). Cold plasma effects on enzyme activity in a model food system. Innov. Food Sci. Emerg. Technol..

[35] Chutia H., Kalita D., Mahanta C., Ojah N., Choudhury A. (2019). Kinetics of inactivation of peroxidase and polyphenol oxidase in tender coconut water by dielectric barrier discharge plasma. LWT.

[36] Wang Y., Zeng L., Deng W., Wang J., Zhang J. (2024). The molecular reactive pathway between lipoxygenase and lipase and reactive species generated in dielectric barrier discharge atmospheric cold plasma: An investigation using molecular docking. Food Chem..

[37] Okyere A.Y., Rajendran S., Annor G.A. (2022). Cold plasma technologies: Their effect on starch properties and industrial scale-up for starch modification. Curr. Res. Food Sci..

[38] Shen C., Chen W., Li C., Cui H., Lin L. (2023). The effects of cold plasma (CP) treatment on the inactivation of yam peroxidase and characteristics of yam slices. J. Food Eng..

[39] Rothwell J., Hong J., Morrison S., Vyas H.K.N., Xia B., Mai-Prochnow A., McConchie R., Phan-Thien K.-Y., Cullen P.J., Carter D.A. (2023). An Effective Sanitizer for Fresh Produce Production: In Situ Plasma-Activated Water Treatment Inactivates Pathogenic Bacteria and Maintains the Quality of Cucurbit Fruit. Microbiol. Spectr..

[40] Lee G.R., Choi S.W., Yoo M., Chang H.J., Lee N. (2023). Effects of Plasma-Activated Water Treatment on the Inactivation of Microorganisms Present on Cherry Tomatoes and in Used Wash Solution. Foods.

[41] Sone I., García Álvarez R., Walsh J., Prabhu L., Sivertsvik M., Noriega Fernández E. (2019). Towards the Next-Generation Disinfectant: Composition, Storability and Preservation Potential of Plasma Activated Water on Baby Spinach Leaves. Foods.

[42] Wang J., Han R., Liao X., Ding T. (2021). Application of plasma-activated water (PAW) for mitigating methicillin-resistant Staphylococcus aureus (MRSA) on cooked chicken surface. LWT.

[43] Yao L., Xu Y., Chen F., Jiang Z., Zhu Y. (2026). Study on the microflora changes and storage quality of chilled fresh chicken treated by cold plasma combined with plasma activated water. LWT.

[44] Chaijan M., Chaijan S., Panya A., Nisoa M., Cheong L.Z., Panpipat W. (2021). High hydrogen peroxide concentration-low exposure time of plasma-activated water (PAW): A novel approach for shelf-life extension of Asian sea bass (Lates calcarifer) steak. Innov. Food Sci. Emerg. Technol..

[45] Thana P., Boonyawan D., Jaikua M., Promsart W., Rueangwong A., Ungwiwatkul S., Prasertboonyai K., Maitip J. (2025). Plasma-Activated Water (PAW) Decontamination of Foodborne Bacteria in Shucked Oyster Meats Using a Compact Flow-Through Generator. Foods.

[46] Vichiansan N., Phanumong P., Mekwilai T., Kumla J., Suwannarach N., Leksakul K., Lumyong S., Boonyawan D. (2024). Effect of plasma-activated water (PAW) on the Shelf lives of whiteleg shrimp (*Litopenaeus vannamei*) and splendid squid (*Loligo formosana*). Food Bioprocess Technol..

[47] Ferreira R.O., Spigarollo D.C.F.S., Silveira Duarte I.C., de Oliveira E.C., da Cruz N.C. (2026). Antimicrobial efficacy and cytotoxic assessment of plasma-activated water generated by a dielectric barrier discharge microbubble system. Front. Microbiol..

[48] Dantas A., Batista J., Lima M., Fernandes F.A., Rodrigues S., Magnani M., Borges G.d.S.C. (2021). Effect of cold plasma on açai pulp: Enzymatic activity, color and bioaccessibility of phenolic compounds. LWT Food Sci. Technol..

[49] Kumar S., Pipliya S., Srivastav P.P. (2023). Effect of cold plasma on different polyphenol compounds: A review. J. Food Process Eng..

[50] Li X., Li M., Ji N., Jin P., Zhang J., Zheng Y., Zhang X., Li F. (2019). Cold plasma treatment induces phenolic accumulation and enhances antioxidant activity in fresh-cut pitaya (*Hylocereus undatus*) fruit. LWT.

[51] Rana S., Mehta D., Bansal V., Shivhare U.S., Yadav S.K. (2020). Atmospheric cold plasma (ACP) treatment improved in-package shelf-life of strawberry fruit. J. Food Sci. Technol..

[52] Gebremical G.G., Tappi S., Laurita R., Capelli F., Drudi F., Romani S., Rocculi P. (2024). Plasma-activated water (PAW) and annealing for the modification of potato starch: Effects on sorption isotherms, thermodynamic and gelatinization kinetics of potato starch. Innov. Food Sci. Emerg. Technol..

[53] Rivero W., Shah U., Wang Q., Zheng H.T., Salvi D. (2024). Exploration of plasma-activated water (PAW) as a cleaning-in-place (CIP) solution for fouling removal and microbial reduction. J. Food Process Eng..

[54] Ghevariya D., Salvi D. (2025). Assessing plasma-activated water as an acidic and sanitizer solution in clean-in-place (CIP) and comparing efficacy with other traditional CIP chemicals. J. Food Sci..

[55] Gülenç B., Özdemir E., Başaran P., Kartal S., Akan T. (2024). Impact on microbiology and product quality of plasma-activated water treatment on fresh green leafy vegetables. Postharvest Biol. Technol..

[56] Hadinoto K., Astorga J.B., Masood H., Zhou R., Alam D., Cullen P.J., Prescott S., Trujillo F.J. (2021). Efficacy optimization of plasma-activated water for food sanitization through two reactor design configurations. Innov. Food Sci. Emerg. Technol..

[57] Royintarat T., Choi E.H., Boonyawan D., Seesuriyachan P., Wattanutchariya W. (2020). Chemical-free and synergistic interaction of ultrasound combined with plasma-activated water (PAW) to enhance microbial inactivation in chicken meat and skin. Sci. Rep..

[58] Liao X., Su Y., Liu D., Chen S., Hu Y., Ye X., Wang J., Ding T. (2018). Application of atmospheric cold plasma-activated water (PAW) ice for preservation of shrimps (Metapenaeus ensis). Food Control.

[59] Herianto S., Shih M.K., Lin C.M., Hung Y.-C., Hsieh C.-W., Wu J.-S., Chen M.-H., Chen H.-L., Hou C.-Y. (2021). The effects of glazing with plasma-activated water generated by a piezoelectric direct discharge plasma system on whiteleg shrimp (*Litopenaeus vannamei*). LWT.

[60] Manoharan D., Stephen J., Radhakrishnan M. (2022). Study on the effect of atmospheric and low-pressure plasma and its combination on the microbial reduction and quality of milk. J. Food Saf..

[61] Nikmaram N., Keener K.M. (2022). The effects of cold plasma technology on physical, nutritional, and sensory properties of milk and milk products. LWT.

[62] Sujatha G., Narayanan R., MSSK (2023). Effect of cold plasma on the quality parameters of custard apple juice milk beverage. Indian J. Dairy Sci..

[63] Pankaj S., Misra N., Alzahrani K., Alamri A., Galanakis C. (2025). Cold Plasma Treatment of Orange Juice Using Multipin-Plane Electrical Discharge. J. Food Process Eng..

[64] Xiang Q., Wang W., Zhao D., Niu L., Li K., Bai Y. (2019). Synergistic inactivation of Escherichia coli O157: H7 by plasma-activated water and mild heat. Food Control.

[65] Moiseienko Y., Shahbaz H., Saad S., Avison M., Stratakos A. (2025). Effect of plasma-activated water against *E. coli* and *S. aureus*: Influence of organic matter and impact on skin cell viability. Appl. Microbiol. Biotechnol..

[66] Fenoglio D., Ferrario M., García Carrillo M., Schenk M., Guerrero S. (2020). Characterization of microbial inactivation in clear and turbid juices processed by short-wave ultraviolet light. J. Food Process Preserv..

[67] Dasalkar A., Biswas A., Chaudhari S., Yannam S. (2025). Effect of UV-C LEDs and heat treatments on microbial safety, chemical and sensory properties of sweet lime juice. Food Chem..

[68] Duering H., Westerhoff T., Kipp F., Stein C. (2023). Short-Wave Ultraviolet-Light-Based Disinfection of Surface Environment Using Light-Emitting Diodes: A New Approach to Prevent Health-Care-Associated Infections. Microorganisms.

[69] Epelle E.I., Macfarlane A., Cusack M., Burns A., Amaeze N., Richardson K., Mackay W., Rateb M.E., Yaseen M. (2022). Stabilisation of ozone in water for microbial disinfection. Environments.

[70] Taiye Mustapha A., Zhou C., Wahia H., Amanor-Atiemoh R., Otu P., Qudus A., Fakayode O.A., Ma H. (2020). Sonozonation: Enhancing the antimicrobial efficiency of aqueous ozone washing techniques on cherry tomato. Ultrason Sonochem..

[71] Nguyen P., Mittal G.S. (2007). Inactivation of naturally occurring microorganisms in tomato juice using pulsed electric field (PEF) with and without antimicrobials. Chem. Eng. Process. Process Intensif..

[72] Yeom H.W., Streaker C.B., Zhang Q.H., Min D.B. (2000). Effects of pulsed electric fields on the quality of orange juice and comparison with heat pasteurization. J. Agric. Food Chem..

[73] Abera G. (2019). Review on high-pressure processing of foods. Cogent Food Agric..

[74] Arnaud C., de Lamballerie M., Pottier L. (2018). Effect of high pressure processing on the preservation of frozen and re-thawed sliced cod (*Gadus morhua*) and salmon (*Salmo salar*) fillets. High. Press. Res..

[75] Garriga M., Grebol N., Aymerich M.T., Monfort J.M., Hugas M. (2004). Microbial inactivation after high-pressure processing at 600 MPa in commercial meat products over its shelf life. Innov. Food Sci. Emerg. Technol..

[76] Beitia E., Gkogka E., Chanos P., Hertel C., Heinz V., Valdramidis V., Aganovic K. (2023). Microbial decontamination assisted by ultrasound-based processing technologies in food and model systems: A review. Compr. Rev. Food Sci. Food Saf..

[77] Mustapha A.T., Zhou C., Wahia H., Sarpong F., Nasiru M.M., Adegbemiga Y.B., Ma H. (2020). Combination of thermal and dual-frequency sonication processes for optimum microbiological and antioxidant properties in cherry tomato. J. Food Process Preserv..

[78] Mustapha A.T., Zhou C., Sun Y., Wahia H., Sarpong F., Owusu-Ansah P., Osae R., Otu P., Ma H. (2019). Simultaneous multifrequency: A possible alternative to improve the efficacy of ultrasound treatment on cherry tomato during storage. J. Food Process Preserv..

[79] Hu Z., Hu Z., Zhang H., Yu Z., Xie Y. (2025). Antimicrobial effects of tannic acid combined with plasma-activated water and their application in strawberry preservation. Foods.

[80] Zhou R., Zhou R., Prasad K., Fang Z., Speight R., Bazaka K., Ostrikov K. (2018). Cold atmospheric plasma activated water as a prospective disinfectant: The crucial role of peroxynitrite. Green. Chem..

[81] Jyung S., Kang J., Kang D. (2023). Inactivation of Listeria monocytogenes through the synergistic interaction between plasma-activated water and organic acid. Food Res. Int..

[82] Roobab U., Madni G., Ranjha M., Khan A.W., Selim S., Almuhayawi M.S., Samy M., Zeng X.-A., Aadil R.M. (2023). Applications of water activated by ozone, electrolysis, or gas plasma for microbial decontamination of raw and processed meat. Foods.

[83] Andreou V., Dimopoulos G., Dermesonlouoglou E., Taoukis P. (2020). Application of pulsed electric fields to improve product yield and waste valorization in industrial tomato processing. J. Food Eng..

[84] Wang B., Wang W., Xiang Q., Bai Y. (2023). Effects of heating on the antibacterial efficacy and physicochemical properties of plasma-activated water. Qual. Assur. Saf. Crops Foods.

[85] Liao X., Bai Y., Muhammad A.I., Liu D., Hu Y., Ding T. (2020). The application of plasma-activated water combined with mild heat for the decontamination of Bacillus cereus spores in rice (Oryza sativa L. ssp. japonica). J. Phys. D Appl. Phys..

[86] Zhou Z., Li H., Qi Z., Liu D. (2024). Biological and chemical reactivities of plasma-activated water prepared at different temperatures. Plasma Chem. Plasma Process..

[87] Wen X., Nie M., Zhang Z., Xiong L., Feng J., Zhang Z., Li D., Bao Y., Wu H. (2025). Effect of Ultrasound Combined with Plasma-Activated Water on Lethal and Sublethal Injury Against Escherichia coli. Foods.

[88] Wu W., Zhang J., Li T., Hu Z., Huang S., Lin M., Xie Y., Yu Z. (2024). Antimicrobial effect of plasma-activated water combined with tartaric acid against Staphylococcus aureus and its application on fresh-cut asparagus lettuce. Food Biosci..

[89] Yoon S., Kim S., Min S. (2025). Sequential treatment of plasma-activated water and in-package cold plasma treatment enhances microbiological safety and storage stability of mung bean sprouts, shrimp, and bok choy mixtures. Int. J. Food Microbiol..

[90] Nikzadfar M., Kazemi A., Abooei R., Firouz M.S., Akbarnia A., Rashvand M. (2024). Application of Cold Plasma Technology on the Postharvest Preservation of In-Packaged Fresh Fruit and Vegetables: Recent Challenges and Development. Food Bioprocess Technol..

[91] Sharma R., Nath P., Rustagi S., Sharma M., Inbaraj B.S., Dikkala P.K., Nayak P.K., Sridhar K. (2025). Cold Plasma—A Sustainable Energy-Efficient Low-Carbon Food Processing Technology: Physicochemical Characteristics, Microbial Inactivation, and Industrial Applications. Int. J. Food Sci..

[92] Li W., Nguyen N.H., Zhou R., Truong V., Cullen P., Vasilev K. (2026). Plug-and-play plasma: Decentralised water decontamination for developing countries. npj Clean Water.

[93] Zou F.L., Yang M., Wu J.H., Wang L.J., Wang H.Y. (2025). The potential of plasma-activated water in safe and sustainable food production: A comprehensive review of recent advances and future trends. Crit. Rev. Food Sci. Nutr..

[94] US Food and Drug Administration (FDA) (2026). Determining the Regulatory Status of a Food Ingredient.

[95] European Parliament and Council of the European Union (2012). Regulation (EU) No. 528/2012 of 22 May 2012 Concerning the Making Available on the Market and Use of Biocidal Products. Off. J. Eur. Union.

[96] European Parliament and Council of the European Union (2004). Regulation (EC) No. 852/2004 of 29 April 2004 on the Hygiene of Foodstuffs. Off. J. Eur. Union.

[97] European Parliament and Council of the European Union (2002). Regulation (EC) No. 178/2002 of 28 January 2002 Laying down the General Principles and Requirements of Food Law, Establishing the European Food Safety Authority and Laying down Procedures in Matters of Food Safety. Off. J. Eur. Union.

[98] Xiang Q., Fan L., Li Y., Dong S., Li K., Bai Y. (2020). A review on recent advances in plasma-activated water for food safety: Current applications and future trends. Crit. Rev. Food Sci. Nutr..

[99] Ricci E., Banterle A., Stranieri S. (2018). Trust to Go Green: An Exploration of Consumer Intentions for Eco-friendly Convenience Food. Ecol. Econ..

[100] Bayati M., Lund M., Tiwari B., Poojary M. (2024). Chemical and physical changes induced by cold plasma treatment of foods: A critical review. Compr. Rev. Food Sci. Food Saf..

[101] Saber K., Hani A., Guedah H., Abahazem A., Merbahi N. (2025). Experimental study and efficiency evaluation of cold plasma treatment for distilled water. J. Plasma Phys..

[102] Andrasch M., Stachowiak J., Schlueter O., Schnabel U., Ehlbeck J. (2017). Scale-up to pilot plant dimensions of plasma processed water generation for fresh-cut lettuce treatment. Food Packag. Shelf Life..

[103] Han J.Y., Min S.C., Lee H.S., Eom S., Ryu S., Cho S., Kang D.-H. (2024). Pilot-scale plasma activated water treatment for decontaminating romaine lettuce and steamed rice cakes. LWT.

[104] Misra N.N., Naladala T., Alzahrani K.J., Sreelakshmi V.P., Negi P.S. (2024). Design of a continuous plasma activated water (PAW) disinfection system for fresh produce industry. Innov. Food Sci. Emerg. Technol..

[105] Sruthi N., Josna K., Pandiselvam R., Kothakota A., Gavahian M., Khaneghah A. (2021). Impacts of cold plasma treatment on physicochemical, functional, bioactive, textural, and sensory attributes of food: A comprehensive review. Food Chem..

